# Diagnostic utility of procalcitonin in inpatients at a community hospital

**DOI:** 10.1002/ccr3.2230

**Published:** 2019-06-03

**Authors:** Nathan T. Douthit, Brytney S. Cobia

**Affiliations:** ^1^ Brookwood Baptist Health Medical Education Birmingham Alabama

**Keywords:** diagnostic testing, infectious disease, laboratory medicine, procalcitonin

## Abstract

Procalcitonin is an important diagnostic tool to be used in conjunction with clinical judgement and other data regarding the infectious etiology and appropriate treatment of the patient's present illness.

## 1 INTRODUCTION

Procalcitonin is a precursor to the hormone calcitonin. It is genetically encoded on the CALC‐1 gene—the expression of which is suppressed in most tissues in noninfectious states.^1^, ^2^ Physiologically, procalcitonin is cleaved into calcitonin, resulting in a low baseline level (<0.05 ng/mL). 1 In bacterial infections, expression of CALC‐1 is upregulated by certain inflammatory cytokines including interleukin‐6 and tumor necrosis factor‐alpha (TNF‐a). In viral infections, a different inflammatory milieu induces cytokines like interferon gamma which inhibit the expression of TNFa, thereby inhibiting procalcitonin production.^2^, ^3^ In light of these findings, procalcitonin is now classified as an acute phase reactant along with established markers like cytokines, leukocytes, C‐reactive protein, and erythrocyte sedimentation rate.^4^ Its utility is enhanced by the fact that it is not commonly elevated in other inflammatory conditions such as rheumatologic disease, and is not affected by steroids.^5^ It has strong correlation with bacterial infection in meningitis, pneumonia, and septic shock; there is moderate data supporting its usefulness in urinary tract, intraabdominal, and neutropenic infections.^5^, ^6^ There is a correlation between the level of procalcitonin and the severity of infection. Levels typically rise between 2 and 4 hours after infection, peak within 24‐48 hours, and fall by 50% per day after the infection is controlled.^7^ False positives can be seen in hypoperfusion, central nervous system processes, tumors with neuroendocrine effects, or renal insufficiency; false negatives are seen in atypical infections or localized infections.^8^

There have been many promising studies describing the clinical utility of procalcitonin, but confusion remains among trainees and experienced clinicians alike on its appropriate diagnostic use. Most institutions have their own guidelines for use and different cutoffs for clinical significance. These primarily focus on antibiotic stewardship.^5^, ^6^ We present four cases in which procalcitonin was measured and interpreted diagnostically on inpatients at a community hospital.

## 2 CASE 1

A 54‐year‐old female patient was admitted from a skilled nursing facility. The patient had a prolonged stay at an outside hospital for pneumonia and respiratory failure requiring mechanical ventilation. She was unable to wean from the ventilator and discharged with a tracheostomy and chronic urinary catheter. At the nursing facility, she became febrile and increasingly somnolent. Vital signs were significant for pulse of 149 beats per minute (bpm), temperature of 105.2°F, saturation of 97%, blood pressure (BP) of 123/85 mm Hg; pertinent laboratories are in Table 1. Physical examination was significant for somnolence, bibasilar crackles and the aforementioned tracheostomy and urinary catheter. Chest X‐ray (CXR) showed bibasilar infiltrates consistent with pneumonia. Vancomycin and piperacillin‐tazobactam were initiated by the emergency department physician (ED‐MD).

**Table 1:** Pertinent laboratories for Patient 1 (abnormal values in bold)

Sodium (mEq/L)	Chloride (mEq/L)	Potassium (mEq/L)	Bicarbonate (mEq/L)	BUN (mg/dL)	Cr (mg/dL)
140	100	4.3	25	**23**	0.86

CBC	WBC/μL	Hemoglobin (g/dL)	Hematocrit (%)	Platelets/μL	MCV (fL)
	**13.3**	**10.2**	**36**	245	**99.4**

Procalcitonin in this patient was normal at 0.31 ng/mL (reference range at our institution <0.50 ng/dL). Regardless, she was continued on broad‐spectrum antibiotics and ETT aspirate grew pseudomonas. The patient improved clinically with antibiotics and was discharged to a skilled nursing facility.

## 3 CASE 2

A 54‐year‐old female patient with a past medical history of chronic obstructive pulmonary disease (COPD) on 4 L/min home oxygen presented after recent discharge from an outside hospital. The patient had been treated for COPD exacerbation with steroids, levofloxacin, and inhaled bronchodilators and was discharged 2 days prior to presentation. She complained of worsening shortness of breath, productive cough, and chills. Vital signs were significant for pulse of 96 BPM, temperature of 98.5°F, saturation of 92%, BP of 140/89 mm Hg; pertinent laboratories are in Table 2. Physical examination was significant for bibasilar crackles, mild peripheral edema, and morbid obesity. CXR showed bibasilar infiltrates which were read as atelectasis, new infiltrates, or pulmonary edema. Vancomycin and piperacillin‐tazobactam were initiated by the ED‐MD for healthcare‐associated pneumonia.

**Table 2:** Pertinent laboratories for Patient 2 (abnormal values in bold)

Sodium (mEq/L)	Chloride (mEq/L)	Potassium (mEq/L)	Bicarbonate (mEq/L)	BUN (mg/dL)	Cr (mg/dL)
139	101	**3.2**	32	**23**	0.91

CBC	WBC/μL	Hemoglobin (g/dL)	Hematocrit (%)	Platelets/μL	MCV (fL)
	**11.2**	12.0	**38**	227	99.0

Procalcitonin was <0.05 ng/mL. She was treated for COPD exacerbation with steroids and inhaled bronchodilators and treated for volume overload with furosemide. She was significantly better after diuresis and discharged home after 2 days with no antibiotics.

## 4 CASE 3

A 37‐year‐old female patient with past medical history significant for polysubstance abuse, COPD, and recurrent admissions for pneumonia presented with shortness of breath and cough. She had regular inhalational methamphetamine use, but states that this had recently been cut with crack cocaine. She thought this may be responsible for her symptoms. Vital signs were significant for pulse of 135 BPM, temperature of 98.3°F, saturation of 56%, BP of 129/83 mm Hg; pertinent laboratories are in Table 3. Physical examination was significant for diffuse expiratory wheezing and scattered inspiratory crackles. CXR showed mild interstitial and peribronchial thickening centrally with mild bibasilar consolidation, which was improved from CXR 1 month prior. Vancomycin and piperacillin‐tazobactam were initiated by the ED‐MD.

**Table 3:** Pertinent laboratories for Patient 3 (abnormal values in bold)

Sodium (mEq/L)	Chloride (mEq/L)	Potassium (mEq/L)	Bicarbonate (mEq/L)	BUN (mg/dL)	Cr (mg/dL)
135	96	3.6	30	8	0.71

CBC	WBC/μL	Hemoglobin (g/dL)	Hematocrit (%)	Platelets/μL	MCV (fL)
	**16.3**	13.4	44	**628**	93.0

Procalcitonin was <0.05 ng/mL. Antibiotics were discontinued, she was treated with steroids and supplemental oxygen therapy for chemical pneumonitis. She substantially improved and left against medical advice on day 3 of her hospitalization.

## 5 CASE 4

A 63‐year‐old male patient presented with pneumonia requiring intubation and mechanical ventilation. During a prolonged hospitalization, he failed extubation multiple times and underwent a brief cardiopulmonary arrest. He received antibiotics for 26/27 hospital days, and his peak procalcitonin was 93.40 ng/dL (Table 4). His procalcitonin was being trended in the interest of antimicrobial stewardship. The patient completed a course of levofloxacin for enterobacter pneumonia on day 27. His procalcitonin on that day was 1.89 ng/dL. It continued to trend down after cessation of antibiotics, indicating control of the infection, until a nadir of 0.49 ng/dL. The patient was managed supportively and eventually discharged to an inpatient rehab facility.

**Table 4:** Course of WBC and PCT during hospital stay

Hospital day	WBC (/uL)	Procalcitonin (ng/dL)
18	9.3	93.40
19	6.5	92.89
21	10.6	19.77
22	14.6	9.49
23	25.7	6.63
24	29.1	3.12
27	20.0	1.89
29	23.1	0.6
30	26.0	0.49

## 6 DISCUSSION

As shown in Case 1, the use of procalcitonin to delay administration of antibiotics has not proven helpful in septic patients, and in some cases has proven harmful.^9^, ^10^ Pretest probability of infection in this patient is high, and the negative procalcitonin is not convincing enough to delay antibiotic treatment. Most institutions use a cutoff of 0.5 ng/dL for critically ill patients and 0.25 ng/dL for stable patients.^5^, ^6^

Cases 2 and 3 show procalcitonin can be a helpful marker in determining whether CXR infiltrates are from bacterial pneumonia or other lung pathology.^11^, ^12^ The use of procalcitonin can help to reduce antibiotic exposure.^13^ This is especially important in these cases as the patients would have needed broad‐spectrum treatment for hospital‐acquired pneumonia.

Finally, Case 4 shows how procalcitonin can be used for antimicrobial stewardship and has helped limit antibiotic exposure in critically ill patients in many cases.^14^ In this patient, the fall and rise of procalcitonin in combination with clinical status helped guide his antibiotic therapy and remove the diagnostic questions of an elevated WBC count in the setting of continued critical illness.

## 7 CONCLUSION

While procalcitonin‐only guided algorithms have not always shown success, its utility as an additional data point in the diagnosis and management of infectious disease is yet to be fully described.^8^, ^10^, ^11^, ^13^ We described four cases where procalcitonin is frequently used to guide care. It should be noted that in the first three of the above patients, initial antibiotic therapy was started appropriately by the ED‐MD, and the test was used along with other clinical factors to decide on the clinical utility of continuing antibiotic therapy. It should also be noted that broad‐spectrum antibiotics were the topic of all 4 of these cases due to their frequent encounters with the healthcare system and consequently, drug resistant pathogens. Procalcitonin should be treated as any other test, with a consideration of pretest probability and how a negative test will impact treatment. It can help differentiate pulmonary pathologies and aid in antimicrobial stewardship. This test can be a valuable tool in the hands of well‐trained clinicians.

## CONFLICT OF INTEREST

None declared.

## AUTHOR CONTRIBUTION

NTD devised the idea for the work and identified patients. Both NTD and BSC were involved in the composing and editing of the manuscript. This was based on a grand rounds given by BSC at our institution.
